# Bullous Dermatosis in an End-Stage Renal Disease Patient: A Case Report and Literature Review

**DOI:** 10.1155/2016/6713807

**Published:** 2016-11-24

**Authors:** Zeenat Yousuf Bhat, Marwan Abu Minshar, Nashat Imran, Andrew Thompson, Yahya Osman Malik

**Affiliations:** ^1^Division of Nephrology and Hypertension, Department of Internal Medicine, Wayne State University Detroit Medical Center, Detroit, MI, USA; ^2^Department of Pathology, Wayne State University Detroit Medical Center, Detroit, MI, USA

## Abstract

Patients with advanced chronic kidney disease including ESRD patients may present with a wide spectrum of cutaneous abnormalities, ranging from xerosis to hyperpigmentation to severe deforming necrotizing lesions. Skin problems are not uncommon in this population of patients, with a clinical presentation that can be quite bizarre, mandating a long list of differential diagnostic possibilities, and subsequent rise of a puzzling diagnostic challenge. We describe an ESRD patient who presented with blistering, nonhealing ulcerative lesions with a diagnostic skin biopsy revealing a mixed pattern of linear IgA bullous dermatosis and dermatitis herpetiformis. A clinical remission could be achieved with pulse intravenous steroids followed by oral maintenance in combination with dapsone, with no evidence of recurrence.

## 1. Case Presentation

We report a 53-year-old African American male, who presented with a 4-week history of gradually worsening painful itchy rash over the extremities and trunk, with subsequent development of blisters, bullae, and vesicles. He has been known to suffer from multiple comorbidities among which are end-stage renal disease (ESRD) presumed to be secondary to diabetic nephropathy (on maintenance hemodialysis since 2012), insulin-dependent diabetes mellitus, systemic hypertension, peripheral neuropathy in addition to advanced peripheral arterial disease (PAD), and chronic osteomyelitis for which he received local wound care. There is also a history of mucoepidermoid carcinoma of left parotid gland (biopsy-proven) in the same year of starting dialysis. There were no new medications, including antibiotics that were prescribed over the three months preceding the presentation. Regular medications included aspirin 81 mgs daily, lisinopril 40 mgs, clopidogrel 75 mgs daily, atorvastatin 20 mgs at night, and calcium acetate 667 mgs three times a day with meals, in addition to IV erythropoietin 8800 units three times a week with dialysis, IV iron sucrose 50 mgs once a week, and IV calcitriol 1 mcg with each dialysis session.

**Figure 1:**
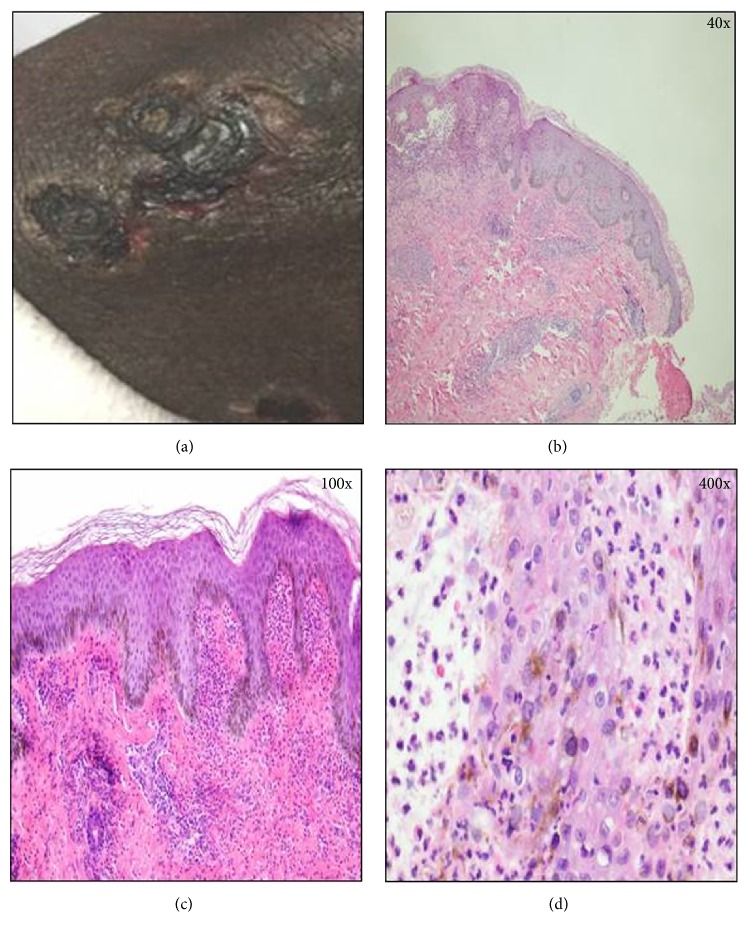
Clinical picture of the bullous lesion along with histopathologic examination of the biopsy specimen. (a) Picture showing bullous lesion on the surface of skin. (b) Histopathologic examination of the specimen shows a punch biopsy specimen Haematoxylin and Eosin (H&E) stain at 40x. Subepidermal vesicle formation is seen with inflammatory infiltrate in the vesicular space. (c) H&E stained specimen at 100x Basket weave stratum cornea is seen overlying epidermis with some exocytosis of neutrophils. (d) H&E staining at 400x. Within the papillary dermis a perivascular infiltrate of neutrophils and lymphocytes with occasional eosinophils is seen.

Physical examination revealed a thin malnourished gentleman weighing 67.8 kgs, bilateral below-knee amputee, pale, but not jaundiced or cyanosed, and in obvious discomfort but fully alert in time, place, and person with no myoclonus or asterixis. Vitals: temperature was 36.3°C, BP was 150/70 mmHg, respiratory rate was 18 per minute, and pulse rate was 84 per minute, thready and regular. Examination of the neck, heart, chest, and abdomen was essentially unremarkable, and clinically he was euvolemic. Peripheral arterial pulses were all absent, and no bruits could be heard over carotids, abdomen, or femoral; however, skin examination revealed extensive bullous lesions over the extremities extensor surfaces and trunk (Figure 1(a)). Many of these lesions had ruptured at different stages to give way to shallow ulcers with a necrotic base. His palm soles, oral cavity, and eyes revealed no evidence of lesions.

## 2. Lab Tests and Other Studies

Serum calcium 8.4 meq/dL, serum phosphorus 7 mg/dL, iPTH 296 pg/mL, other electrolytes within normal limits including bicarbonate of 25 meq/L, blood urea nitrogen (BUN) 59 mg/dL, serum creatinine 7.22 mg/dL, most recent percentage urea reduction ratio 83%, serum albumin 2.1 g/dL, 25 hydroxy-vitamin-D 14 ng/mL, random serum aluminum < 10 ng/mL, Hb 11.0 gm/dL, and WBCs 4.7 K/Cumm, with normal differential count, platelets 237000 K/Cumm, and C-reactive protein > 190 mg/mL. Serology: hepatitis B and hepatitis C were negative; anti-nuclear anti-body, anti-neutrophil cytoplasmic anti-body, and mycoplasma IgM were all negative; herpes simplex virus was positive for both IgM and IgG. Serum protein electrophoresis was consistent with hypoalbuminemia and no monoclonal spikes were present. Serum immunofixation was negative. Tissue transglutaminase IgA level was within normal limits (10 Z units), and glucose-6 phosphate dehydrogenase (G-6PD) level was 14.2 (9.9–14.2/gmHb). The celiac screen was negative.

## 3. Hospital Course

Upon admission, the initial clinical impression was that of a combination of both calciphylaxis (CUA) and extensive peripheral vascular disease. He was discharged with plans to see dermatologist, along with wound care and hyperbaric oxygen therapy as outpatient. One week later, he was rehospitalized with more itchy and ulcerative lesions associated with severe pain. There was no clear exposure to any medication such as vancomycin, furosemide, or allopurinol making the index of suspicion for a diagnosis of bullous drug-induced dermatosis such as linear IgA bullous dermatosis (LABD) or dermatitis herpetiformis quite low at the time of presentation. Based on the clinical probability of CUA, a decision was made to start him on the standard therapy along those lines (wound care, hyperbaric oxygen, and intravenous-sodium thiosulphate, in addition to strict phosphate control and intensive hemodialysis), while an urgent dermatology consultation was sought in the meantime. However, the clinical response to the above regimen was minimal with persistent symptoms, until a decision was made to add IV pulse steroids. A remarkable subjective as well as an objective improvement was noticed, following which the biopsy results became available.

A skin biopsy from the lesions was done by dermatologist. Examination of the sections cut from the frozen tissue block by direct immunofluorescence microscopy (photograph not taken at the time of slide immunofluorescence) showed granular deposition of IgA in dermal papillae. Weak granular deposition of C3 and some deposition of fibrinogen in dermal papillae were also seen. Deposition of fibrinogen was seen in a vascular pattern in papillary dermis that was suggestive of dermatitis herpetiformis versus linear IgA bullous dermatosis (LABD), with no evidence of calciphylaxis (Figure 1). No significant deposition of IgG or IgM was seen. Based on the above biopsy findings, intravenous methylprednisolone was followed by oral prednisone, with oral dapsone, and sodium thiosulphate was discontinued. He was kept on antibiotics (daptomycin for treatment of osteomyelitis and cefepime for the secondarily infected skin lesions). Also, he underwent right hand digits amputation for worsening ischemic gangrene.

There was complete resolution of pruritus and cutaneous lesions that was achieved by the third week of therapy with steroids and dapsone. This therapy was continued for 2 months, with slow taper of steroids over few weeks. There was no recurrence of the lesions.

## 4. Discussion

The skin lesions reported in ESRD patients are diverse and difficult to diagnose clinically on the bedside only [1]. While some lesions are benign, others can be bullous with subsequent ulceration, causing severe morbidity and mortality. Among bullous disorders, IgA-related conditions such as linear immunoglobulin A (IgA) dermatosis (LABD) and dermatitis herpetiformis (DH) are less common autoimmune dermopathies, characterized by subepidermal vesiculobullous erosive mucocutaneous lesions, with different patterns of IgA deposition in dermal papillae and the basement membrane zone (BMZ).

IgA deposition leads to a complex interplay of both humoral and cell mediated immunological processes that result in complement activation, neutrophil, and other mononuclear cell chemotaxes. This eventually leads to loss of adhesion with disruption of the dermal-epidermal junction resulting in blister formation. Although no specific triggering factors have been identified, infections and drugs have been implicated as possible inciting factors. In LABD patients the most common antigen target of Ig A1 is one of the fragments of the extracellular portion of bullous pemphigoid antigen 2 (BP180), 97-KDa or 120 KDa [2, 3]. Genetic predilection has also been suggestive of an association with HLA B8, DR3, CW7, and TNF-2 alleles among others for LABD and almost all patients with DH carry the HLA DQ2 or HLA DQ8. Non-HLA and environmental factors have also been implicated.

We are not aware if there is any clear association between ESRD and development of LABD and DH. After conducting an extensive literature review, we only came across a few sporadic case reports on LABD and only a single case report of DH in an ESRD patient. As a separate entity LABD was first reported by Chorzelski et al. in 1979 [4], in a patient with normal renal function, followed by a cluster of reports and small case series in the past 3 decades, the largest of which was by Kuechle et al. [5] who have reported six cases of drug-induced LABD, 50% of which were after CABG, while the remainder were sporadic (sepsis, malignancy, and seizures), who were given a wide spectrum of medications [5–7] (see the following list).

*Medications Associated IgA Dermopathy*

Atorvastatin
Vancomycin
Co-Trimoxazole
Phenytoin
Penicillin Compounds
Interleukin-2
Lithium
Amiodarone
Captopril
NSAIDs
Rifampin
Furosemide

This reasonably strong association with drugs might be explained by the polypharmacy ESRD patients generally receive, making drug-induced LABD more prevalent as a result. Joly et al. [8] were the first to report a case of ESRD on HDx with DH in 1987, which was thought to be idiopathic. In addition to association with drugs, the two IgA dermopathies have been linked to both autoimmune conditions (thyroid disease, IDDM, SLE, atrophic gastritis, and pernicious anemia) and malignancies (non-Hodgkin's Lymphoma/Hodgkin's Lymphoma) (see* Medications Associated IgA Dermopathy*) [9–11]. The interaction of diabetes, infection, malignancy, vascular disease, and numerous autoimmune conditions (see the following list) in LABD as well as DH could be from the fact that they have common HLA genetic background.

*Disease Associated IgA Dermopathy*

Lymphoproliferative disorders
Esophageal cancer
Systemic lupus erythematosus
Rheumatoid arthritis
Sjogren's syndrome
Ulcerative colitis
Crohn's disease
Thyroid disease
Atrophic gastritis
Insulin-dependent diabetes mellitus

DH, another IgA-related dermopathy, poses quite a significant diagnostic dilemma, both histologically and clinically. DH may be confused with numerous other conditions because of its pleomorphic manifestations and the occasional lack of diagnostic lesions. Differential clinical diagnosis includes erythema multiforme, neurotic excoriations, scabies, eczema, popular urticarial, pemphigoid, and most importantly LABD which is quite difficult to differentiate clinically and histologically. The bedside clinical differentiation between DH and LABD is quite difficult as both conditions share the same clinical presentation with minor differences which could easily be missed by the unwary. For instance, mucosal involvement is unknown in DH while it is well-reported in LABD. In addition bullous lesions are much larger in LABD compared to DH [12–14]. While drug-induced IgA dermopathy is only reported with LABD, it is almost unheard of in DH. On the other hand, gluten-sensitive enteropathy is strongly associated with DH (up to 70%) with quite a rare occurrence in LABD [15]. Although DH and LABD can resemble each other closely with the histopathological features of blisters in LABD and DH almost indistinguishable, their immunological features can be distinctive in both entities. In intact skin, especially adjacent to the lesions, granular IgA deposits are present, while the homogenous linear-band appearance of IgA in BMZ is almost pathognomonic of the diagnosis of DH and LABD, respectively [16, 17].

In our particular patient who has severe PAD and long-standing secondary hyperthyroidism (2HPT) we think the diagnosis falls under an overlapped picture between both LABD and DH. Multifactorial suspicious triggering factors might have played a role in development of this condition including atorvastatin therapy, parotid NHL-tumor, IDDM, and chronic infection.

Given the rarity (<0.5 to 2.3 cases per million individuals per year), the therapy of the 2 conditions is not well defined and is based on only clinical observations, case series, and case reports. Although dapsone remains the first-line therapeutic agent its use may be limited by the unfavorable side effect profile, and another first-line agent is the systemic steroid therapy. High dose steroids for LABD can be used initially to accelerate the improvement and can also be used in dapsone nonresponders/failures. Other agents that have been used are calcineurin inhibitors and mycophenolate. There are a few case reports of intravenous immunoglobulin with promising results in refractory cases [18]. The second-line therapy for dapsone intolerance includes sulfamethoxypyridazine, sulfapyridine, and colchicine [19]. Tetracycline with or without nicotinamide and immunoadsorption has also shown some benefit [20, 21]. In the drug-induced form the corner stone of the treatment is withdrawal of the offending agent.

The response to therapy in the two conditions is not identical. While DH might take a longer time to show improvement to single therapy with dapsone, LABD responds much faster to regimens involving steroids, which combat the intense inflammatory response that accompanies blister formation. In our patient the rapid therapeutic response to steroid therapy is highly suggestive of a major underlying LABD component rather than pure DH [12, 22]. Although clinical and histological presentations can be quite variable, LABD and DH should be considered as a diagnostic possibility when an ESRD patient presents with a bullous eruption. To our knowledge, the combination of severe PAD, 2HPT, and ulcerative bulbous lesions in an ESRD diabetic patient on HD therapy has not been reported in the past.

In our patient, the possibility of a more broadly labelled IgA-related dermopathy as an initial clinical diagnosis was not high in our list of differential diagnoses. This is largely explained by the high probability of CUA, as our patient has multiple risk factors for this condition rather than LABD or DH. This is also confounded further by the severe PAD and severe digital ischemia, which can lead to severe distal ulceration. The lack of significant response to standard CUA management was highly suggestive of an underlying alternative diagnosis.

## 5. Conclusion

Cutaneous lesions affect up to 50% of ESRD patients, many of which are quite disabling and associated with significant morbidity and mortality. The interaction between IgA-associated dermopathies and ESRD appears quite complex. An accurate tissue diagnosis is required in the majority of cases to guide therapy. While a skin biopsy is awaited, a bedside clinical diagnosis is critical in guiding the therapeutic plan in ESRD patients who present with new-onset cutaneous lesions. The probability of DH/LABD should be entertained as one of the major differential diagnoses in ESRD patients presenting with a new-onset bullous rash. In conclusion early diagnosis and treatment of this condition are very essential, as appropriate therapy will reduce subsequent morbidity and mortality.
